# Recurrent Small Bowel Obstruction Following Combined Use of Clozapine and Tirzepatide: A Case Illustrating Additive Gastrointestinal Hypomotility

**DOI:** 10.1192/bjo.2026.11778

**Published:** 2026-06-30

**Authors:** Mariana Galvao de Oliveira, Melisa Kanber

**Affiliations:** South London and Maudsley NHS Foundation Trust, London, United Kingdom

## Abstract

**Aims:**

**Aims (Background)**

Clozapine is associated with severe gastrointestinal hypomotility, constipation, ileus, and bowel obstruction through anticholinergic and antiserotonergic mechanisms. Tirzepatide, a dual glucose-dependent insulinotropic polypeptide (GIP) and glucagon-like peptide-1 (GLP-1) receptor agonist, delays gastric emptying and intestinal transit as part of its pharmacodynamic effect. Both agents independently impair gastrointestinal motility, yet their combined use is increasing as tirzepatide is prescribed to manage clozapine-associated metabolic dysfunction.

We hypothesised that the pharmacological combination of clozapine and tirzepatide may produce additive gastrointestinal hypomotility, increasing the risk of small bowel obstruction (SBO) in vulnerable patients.

**Methods:**

**Methods (Case Report)**

We describe a single case (n=1) of a 54-year-old Black British woman with paranoid schizophrenia treated with clozapine since 2016, type 2 diabetes mellitus, and chronic constipation despite regular laxative therapy. Her medical history included extensive abdominal surgery for sigmoid volvulus with subtotal colectomy and ileostomy in 2012, followed by reversal in 2013.

Tirzepatide was initiated in March 2025 by the diabetes team for poor glycaemic control. Weight, glycaemic indices, clinical presentation, and radiological findings were reviewed using electronic health records.

**Results:**

**Results (Discussion)**

Following initiation of tirzepatide, significant metabolic improvement occurred, with weight reduction from 71.5 kg to 64 kg and Hbs-782c improvement from 86 mmol/mol to 43 mmol/mol within four months.

In May 2025, approximately two months after starting tirzepatide, the patient was admitted with acute abdominal pain and vomiting. Computed tomography (CT) imaging demonstrated markedly dilated small bowel loops (up to 63 mm) with an abrupt transition point, consistent with SBO. A second SBO occurred in October 2025, again confirmed radiologically with dilated loops and a transition point, without evidence of malignancy, perforation, or ischaemia.

There was no documented history of SBO in the years preceding tirzepatide initiation. Both episodes were managed conservatively. Tirzepatide was discontinued following the second obstruction.

**Conclusion:**

**Conclusion**

This case highlights a potential pharmacodynamic interaction between clozapine and tirzepatide, resulting in additive gastrointestinal hypomotility and recurrent SBO in a high-risk patient. While tirzepatide provided substantial metabolic benefit, its use alongside clozapine may pose significant gastrointestinal risk. Careful patient selection, baseline bowel assessment, and close monitoring are essential when prescribing GLP-1/GIP agonists to individuals receiving clozapine.

